# The Role of Pelvic Lymphadenectomy in the Management of Prostate and Bladder Cancer

**DOI:** 10.1100/tsw.2007.148

**Published:** 2007-03-02

**Authors:** Michael E. Woods, Michael Ouwenga, Marcus L. Quek

**Affiliations:** ^1^ Department of Urology, Tulane University Health Sciences Center, 1430 Tulane Avenue, SL-42, New Orleans, LA 70112, USA; ^2^ Department of Urology, Loyola University Stritch School of Medicine, 2160 South First Avenue, Building 54, Room 261, Maywood, IL 60153, USA

**Keywords:** prostate cancer, bladder cancer, lymphadenectomy, lymph node dissection

## Abstract

A pelvic lymph node dissection is commonly performed by urologists in the surgical management of prostate and bladder cancer. Identification of lymph node metastases provides important prognostic information for both diseases. Despite advances in radiographic imaging, a pelvic lymphadenectomy remains the most accurate method to stage lymph node involvement. In the past two decades, there has been an increase in the diagnosis of early stage prostate cancer, which has led some to omit a pelvic lymphadenectomy in patients thought to have low probability of positive lymph nodes. There is little debate, however, over the inclusion of a lymph node dissection in bladder cancer given the approximately 25% incidence of unsuspected nodal disease at the time of surgery. Controversy exists over the extent of an appropriate lymphadenectomy and its therapeutic efficacy. This review will examine the need, extent, and the potential prognostic and therapeutic benefits of a pelvic lymphadenectomy in prostate and bladder cancer.

## INTRODUCTION

Regional lymphadenectomy in addition to primary tumor excision has been incorporated into the routine treatment of many malignancies[1,2,3]. In genitourinary surgery this is clearly seen in the management of testis and penile cancer. The role of pelvic lymphadenectomy in prostate and bladder cancer has not yet been defined to the same extent. Given the variability of lymph node metastases and low sensitivity of current imaging modalities, lymphadenectomy remains the only method to reliably stage prostate and bladder cancer patients[4,5,6]. Accurate lymph node staging cannot be overstated in either disease due to its prognostic implications and consequent identification of those who might benefit from subsequent adjuvant therapies[6,7,8]. Despite the important prognostic information gained from a pelvic lymphadenectomy, there exists some debate over the extent of dissection necessary, potential therapeutic benefit involved, and, in the case of prostate cancer, whether a pelvic lymphadenectomy can be safely omitted in select patients. The remainder of this review will focus on the current status of pelvic lymphadenectomy in prostate and bladder cancer.

## PELVIC LYMPHADENECTOMY IN PROSTATE CANCER

Prostate cancer is the most common cancer, other than skin cancer, in American men. The American Cancer Society (ACS) estimates that during 2006 over 234,000 new cases of prostate cancer will be diagnosed in the United States. About 1 man in 6 will be diagnosed with prostate cancer during his lifetime, and 1 man in 34 will die of it. Currently over 1.8 million men in the United States are survivors of prostate cancer.

The role of lymphadenectomy in the management of prostate cancer remains a controversial issue. It is generally accepted that lymphadenectomy is helpful in staging, but the therapeutic advantage of lymphadenectomy has been debated. Other controversial issues center on the indications for lymphadenectomy, the recommended extent of dissection, and the appropriate number of nodes to be sampled.

## INDICATIONS FOR LYMPHADENECTOMY

With regard to the necessity of lymphadenectomy at the time of radical prostatectomy, many physicians rely on nomograms or risk stratification strategies based on preoperative biopsy results, serum prostate-specific antigen (PSA) levels, and clinical stage. According to these nomograms, patients with a PSA less than 10, biopsy Gleason score less than 7, and clinically localized disease have a low incidence of lymph node involvement and therefore, lymphadenectomy may not be necessary[9,10]. These nomograms, however, are based primarily on series in which limited lymph node dissections were performed. As such, they may understage patients, whereas an extended lymphadenectomy by definition, samples more nodes and can more often detect occult disease[11]. Burkhard et al.[12] found up to 7% of these "low risk" patients (clinically localized disease, Gleason sum <7 and PSA <10.0) to have positive nodes. Additionally, up to one third of prostate biopsies understage prostate cancer, therefore it is sometimes difficult to accurately identify those who do in fact have low risk disease. These limitations in the nomograms and accuracy of prostate needle biopsies have led some experts to recommend lymphadenectomy in all patients undergoing prostatectomy[11].

Traditional imaging modalities (CT and MRI) have demonstrated relatively low sensitivity in the detection of involved lymph nodes[4]. Newer modalities including MRI with intravenous administration of lymphotropic superparamagnetic nanoparticles may improve the sensitivity, but these newer modalities await further investigation[13]. Until another technique is shown to be equally or more sensitive at detection of lymph node metastases, pelvic lymph node dissection remains the most accurate method to stage patients undergoing radical prostatectomy[11].

In addition, the accuracy of standard histopathologic assessment of the retrieved lymph nodes has recently been questioned by Pagliarulo and colleagues[14]. This group reevaluated all of the nodes from 180 radical prostatectomy specimens using immunohistochemical methodology. A total of 24 of the 180 patients (13.3%) with an initial diagnosis of pT3N0 disease were found to have occult nodal involvement when reexamined immunohistochemically. Therefore, until more sensitive methods such as immunoassays are routinely implemented, standard histologic examination may pathologically understage a significant number of patients. It is these patients with low volume micrometastatic disease, in particular, that may potentially benefit from a meticulous pelvic lymphadenectomy.

## TEMPLATE FOR LYMPHADENECTOMY

The lymphatic drainage of the prostate is variable, with the primary landing site situated along the external iliac vein and obturator fossa. Although based on a lymphoscintigraphy study, approximately 20% of positive lymph nodes are primarily along the internal and external iliac artery[15]. The template for a limited pelvic lymph node dissection for prostate cancer typically involves removal of the nodes along the medial aspect of the external iliac vein from the node of Cloquet to the bifurcation of the common iliac vein, as well as the nodes in the obturator fossa anterior to the obturator nerve. Extended lymphadenectomy adds the area below the obturator nerve, and the tissue along the hypogastric artery[11]. Others have also included presacral lymph nodes within an extended dissection[11,16].

## NUMBER OF NODES TO BE REMOVED

In a study by Weingartner, pelvic lymphadenectomy was completed on cadavers without prostate cancer as well as a group of patients with clinically organ-confined prostate cancer. The results demonstrated that the mean number of lymph nodes removed using an extended lymphadenectomy template was 22.7 in the cadaver group versus 20.5 in the patient group[17]. In a report by Burkhard et al.[11] of 365 consecutive patients undergoing extended pelvic lymphadenectomy, a median of 21 nodes were removed. In a study comparing laparoscopic lymphadenectomy with a standard versus an extended template, nearly twice as many lymph nodes were removed in the extended template group (9.3 vs. 17.8)[18]. Similar results were seen in an open prostatectomy series by Heidenreich[16] in which the extended group had a median of 28 nodes compared to the standard group where 11 nodes were removed. There is no consensus on the absolute number of nodes to be removed, but an extended template will typically yield higher node counts. It has been advocated by some that at least 13-20 lymph nodes be removed in a pelvic lymphadenectomy for prostate cancer to achieve accurate staging[17,19].

## RATIONALE FOR EXTENDED PELVIC LYMPHADENECTOMY

In a study by Bader et al looking at 365 pelvic lymphadenectomy specimens, with a negative metastatic evaluation and a mean PSA of 11.9, 24% of the patients were found to have positive nodes. In those patients with positive nodes, 58% were positive along the internal iliac artery. More importantly, in 19% of those with positive nodes, the internal iliac nodes were the only nodes positive for disease[20]. Without a node dissection that includes the nodes along the internal iliac vessels (i.e. extended lymphadenectomy) over half of the patients would have positive nodes remaining, and nearly 20% would be understaged. Tenaglia et al.[21] added support to these finding in a prospective series of 273 patients where the internal iliac nodes were involved 48% of the time, and found exclusively in the internal iliac nodes 15% of the time. In a large retrospective series of over 5,000 prostatectomies by Masterson et al.[22], the authors demonstrated improved staging with increasing node counts as they experienced essentially a linear relationship between the number of lymph nodes removed and the probability of finding positive nodes. This finding has been confirmed by several other groups[16,19,23,24]. The importance of appropriate staging cannot be overstated given the potential affect node positive status may have on consideration for adjuvant therapies. This is based on the landmark work of Messing et al.[7] that demonstrated a survival benefit to immediate versus delayed hormonal therapy in men with lymph node metastases.

With such data, one may wonder why extended pelvic lymphadenectomy has not been widely adopted. There is currently a single prospective randomized trial comparing limited versus extended lymphadenectomy. In this study, 123 patients with clinically localized prostate cancer were assigned to a standard template on one side, and an extended template on the contralateral side. Pelvic lymph node metastases were found on the side of the extended dissection in 4 patients, on the side of the standard template in 3, and on both sides in 1 patient. Interestingly, this study demonstrated a complication rate nearly 3 times higher on the side of the extended dissection. The authors[25] suggest that extended lymphadenectomy identifies few patients with nodal disease that would not otherwise be discovered with a more limited dissection, and that extended lymphadenectomy may have a higher associated complication rate[25]. DiMarco et al.[26] published a retrospective study evaluating the outcomes of patients after radical prostatectomy based on the extent of lymph node dissection and found no difference in PSA progression or disease-specific mortality.

## MORBIDITY OF PELVIC LYMPHADENECTOMY IN PROSTATE CANCER

Typical complications associated with lymphadenectomy include lymphoceles, lymphedema, pelvic abscess, venous thrombosis, and pulmonary embolism. The complication rate for pelvic lymphadenectomy for prostate cancer has been reported to occur in 4-50% of patients historically[27]. Although a contemporary series reported around a 10% incidence[25]. In a study by Heidenreich[16], there was no difference in intraoperative (8.7 vs 9.0%) or postoperative (26.2 vs 27.0%) complications between patients undergoing an extended versus limited lymphadenectomy. Another study comparing an extended to limited staging lymph node dissection reported a 36 vs 2% complication rate[18]. These results should be interpreted with caution. This was a laparoscopic series and there were only 40 patients in the extended group versus 150 in limited group. Some techniques that have been suggested to reduce the complication rate include using ligatures instead of hemoclips, as they may tend to tear away during further surgery. If low-molecular weight heparin is used, some advocate injection into the upper extremity. Venous thrombosis and embolism can be minimized by limiting the use of electrocautery near the major pelvic veins. Also, placement of a surgical drain in each side of the pelvis, and not removing these drains prematurely may prevent postoperative lymphoceles[11]. Although there is scant data to evaluate the difference in complications between an extended and limited dissection, the authors believe if sound surgical principles are followed an extended lymphadenectomy can be safely performed without increased risk in the appropriate patient.

## IS THERE A THERAPEUTIC BENEFIT TO LYMPHADENECTOMY?

In malignancies such as gastric, breast, colon, and bladder cancer, it has become apparent that lymphadenectomy not only improves staging but also impacts survival[11]. In prostate cancer, it is accepted that pelvic lymphadenectomy is useful in staging, but the therapeutic benefit of lymphadenectomy remains controversial. In the Messing[7] study, of those patients randomized to the observation group, 16% did not display any indication of PSA recurrence at a median follow-up of 7.1 years. The extent of lymphadenectomy was not described, nor was this the aim of that study, although it does appear that removal of the diseased nodes may have had a therapeutic benefit in this small subset of patients. These findings were supported by Palapattu et al.[28] in a report on 3,264 consecutive prostatectomies with extended pelvic lymphadenectomy. In this series, 143 (4.4%) were found to pelvic lymph node involvement. Within this group of patients, 24 (16.8%) were free of disease at a median follow-up of 6 years[28].

In a recent series of 235 patients with node positive prostate cancer, those who had 1 or 2 positive lymph nodes had a clinical recurrence-free survival of 70% and 73% at 10 years, respectively, versus 49% in those who had 5 or more involved lymph nodes[29]. The 10-year overall recurrence-free survival for this cohort was 65%, although 31% of these patients received adjuvant therapy. In another study by Bader et al, 92 of 367 patients were found to have nodal involvement at the time of prostatectomy for clinically localized disease. Of the 39 patients with only 1 positive node, 15 (39%) remained without signs of clinical or biochemical progression. Of those with 2 positive nodes, only 2 (10%) remained disease-free[23]. Allaf et al.[24] demonstrated improved 5-year PSA-free rate in node positive patients with a lymph node density (number of positive nodes divided by total number of nodes removed) less than 15% who underwent an extended versus limited lymph node dissection, 43 vs 10%. Lymph node density was also shown by Daneshmand et al.[29] to define patients at risk for PSA recurrence. These authors used a node density cutoff of less than 20% versus 20% or greater and demonstrated a 10-year recurrence free survival of 72% versus 47%, respectively.

An improved survival has also been suggested in node-negative patients undergoing extended lymphadenectomy. In an analysis of the SEER (Surveillance, Epidemiology, and End Results Program) database, Joslyn and Konety[30] found that N0 patients with 10 or greater lymph nodes had a significantly lower cancer specific mortality. Obviously some of the benefit seen within these studies may arise from more accurate staging. These studies do suggest, however, that removing a greater number of lymph nodes, which may harbor micrometastatic disease, can provide some therapeutic advantage for select patients.

## PELVIC LYMPHADENECTOMY IN BLADDER CANCER

Urothelial carcinoma of the bladder is the fifth most common malignancy in the United States. It is estimated during 2006 that approximately 61,420 new cases of bladder cancer will be diagnosed and nearly 13,060 deaths will result from the disease[31]. Most cases are superficial at diagnosis, although 20-40% will present with or progress to muscle invasion[32]. Radical cystectomy remains the standard therapy for clinically localized bladder cancer with well-documented oncologic outcomes[6,32].

Despite improvements in radiographic imaging, about a quarter of patients will have pathologic evidence of lymph node metastases at the time of radical cystectomy[6,33,34,35]. Since patients with lymph node involvement are known to have worse survival rates,[6,35,36,37] accurate pathologic staging of these patients will identify those who may benefit most from adjuvant therapy. In addition, some node positive patients achieve long-term disease-free survival from cystectomy and pelvic lymphadenectomy alone, suggesting the procedure may be curative in select patients[6,36,38,39,40].

## TEMPLATES FOR LYMPHADENECTOMY IN BLADDER CANCER

Skinner[41] described the template for an “extended” lymph node dissection. The distal extent is the circumflex iliac vein and lymph node of Cloquet, including all lymphatic tissue posterior to the obturator nerve lying between the pelvic sidewall and the bladder and rectum. The lateral extent is the genitofemoral nerve bilaterally. Bilateral presciatic and presacral tissue is included. The cephalad extent of the extended dissection is the inferior mesenteric artery (IMA), including distal paracaval and paraaortic tissue. A “standard” dissection template shares similar distal and lateral borders but the cephalad extent is typically up to the bifurcation of the iliac vessels and presacral tissue is not routinely included.

## INCIDENCE OF NODE POSITIVE BLADDER CANCER

The incidence of lymph node metastases in patients undergoing radical cystectomy ranges from 14-32.4%[6,35,37,42,43,44,45,46]. Increasing primary tumor stage is clearly associated with an increased risk of lymph node involvement. The incidence of lymph node metastases for non-invasive (Tis,Ta,T1), T2, T3, and T4 disease have been reported to be 1.8-9.6%, 15.6-19.4%, 38.8-49%, and 42-75%, respectively[6,35,37,42,44,46].

A multi-center, prospective study from Leissner and colleagues[44] evaluated the lymphatic spread and distribution of metastases in lymph node positive patients. Even for unilaterally-confined primary tumors, contralateral spread was found at all levels[44]. Although a unilateral dissection or sentinel node excision has been advocated in the past[45], this study supports the importance of routinely performing a bilateral dissection, regardless of primary tumor location[33,39]. This study also classified lymph node metastases into three levels-distal to the bifurcation of the iliac vessels, between aortic bifurcation and bifurcation of the iliac vessels, and between the IMA and the bifurcation of the aorta. Based on this classification, if the dissection was performed in “standard” fashion (proximal extent being the bifurcation of the common iliac vessels), 6.9% of patients would not have been diagnosed with node positive disease and 43.7% of all positive lymph nodes would have been left behind. Of all patients with a single positive node, 10% were located above the bifurcation of the common iliac vessels.

Vazina et al.[46] reported a retrospective study evaluating the incidence and location of lymph node metastases by pathologic T-stage. The incidence of positive lymph nodes did increase with T stage, but involvement of common iliac, presacral, and distal paraaortic nodes was seen in all T-stages (T2-T4). This study lends additional support that an extended lymphadenectomy may benefit patients undergoing radical cystectomy regardless of the extent of the primary tumor.

## FACTORS AFFECTING LYMPH NODE COUNTS DURING PELVIC LYMPHADENECTOMY

Just as with prostate cancer, several factors play a role in the absolute number of lymph nodes evaluated in lymphadenectomy specimens following radical cystectomy. These factors are related to inherent patient variability, pathologic evaluation, surgeon characteristics, as well as the template used for the dissection[47].

The role of the pathologist is central in lymph node identification. The diligence to locate small, grossly negative nodes will vary among and within institutions. The effect of submitting specimens en bloc or in separate packets has been evaluated prospectively. It was demonstrated that lymph node counts increased from 2.4 to 8.5 in a standard template and from 22.6 to 36.5 lymph nodes with an extended template when the specimens were submitted as separate packets as opposed to an "en bloc" fashion[48]. The authors concluded that with bulky en bloc specimens it was more likely that only palpable or grossly positive nodes would be identified. However, with individual, less bulky packets, it was easier for the pathologist to dissect and identify smaller lymph nodes.

The boundaries of dissection have a significant impact on lymph nodes counts. In a multicenter study by Herr et al.[47] the number of lymph nodes removed increased from 13 to 26 when an extended dissection was performed. Poulsen et al.[42] found that by extending their dissection to the bifurcation of the aorta, the average lymph node counts increased from 14 to 25. This was also reported by Bochner who experienced a greater than four-fold increase in lymph nodes removed (8.5 to 36.5) using an extended dissection[48]. Similar results were reported by Gill and coworkers with laparoscopic pelvic lymphadenectomy, in which extension of the dissection to the proximal common iliacs, increased the number of lymph nodes removed from 3 to 21[49]. Since adopting the "extended" template and routinely submitting individual lymph node packets to the pathologists, we have also noted a greater than 5-fold increase in average node counts from 10 (range: 1-44) to 55 (range: 26-105) (unpublished data).

## HOW MANY LYMPH NODES SHOULD BE REMOVED?

There is no consensus on the minimum number of lymph nodes that should be removed during lymphadenectomy for bladder cancer, although several groups have made recommendations on the minimum number that should be evaluated. Leissner et al.[35] stated that removal of 20 lymph nodes would be a reasonable goal. This was based on the fact that when <16 lymph nodes were removed, 60% of node positive patients were identified, whereas if 20 lymph nodes were removed, then 80% of node positive patients were included. A large retrospective, multi-center study was conducted to evaluate radical cystectomy and pelvic lymph node dissection in attempt to develop pathologic goals for the procedure[47]. The study group included 4 high volume institutions and concluded that 10-14 lymph nodes should be removed during pelvic lymphadenectomy. A similar recommendation was made by Konety et al.[32] in a retrospective review of the SEER database (Surveillance, Epidemiology, and End Results program) to determine the effect of lymph node dissection on the outcome of patients undergoing radical cystectomy. This study involved many institutions and surgeons and was subsequently more heterogeneous then the previous study[47].

Herr analyzed the surgical factors within a multi-institutional prospective study designed to evaluate the role of neoadjuvant chemotherapy. The study enrolled 317 patients who were randomized to cystectomy alone or chemotherapy followed by cystectomy[50]. Two hundred and seventy patients underwent cystectomy. There was variability in the extent of lymphadenectomy, with 24 patients receiving no dissection at all. The survival was significantly different between patients with >10 versus

<10 lymph nodes removed. Further analysis of surgical factors demonstrated that the extent of lymph node dissection, number of lymph nodes removed, and surgeon experience were significant factors affecting survival. He hypothesized that increased node counts resulted in improved survival for the following reasons: a diminished risk of local and regional recurrence by removal of micrometastatic disease, more complete dissection with wider margins, and a more thorough evaluation by the pathologist that can improve staging. This report highlights the role of surgery in the comprehensive management of muscle-invasive bladder cancer.

## MORBIDITY OF PELVIC LYMPHADENECTOMY

Modern anesthetic and perioperative care, along with improved surgical technique, have greatly reduced the mortality rate of patients undergoing radical cystectomy. Despite these improvements, overall complication rates are still significant. This patient population is at particularly high risk for postoperative complications given their age and commonly associated co-morbidities. An extended lymph node dissection may provide improved staging accuracy and survival, but does it cause any additional morbidity and mortality? Mortality rates of 1-2.5% and complication rates of 17-28% have been reported in radical cystectomy series using extended lymph node dissections[6,33]. In a recent analysis of mortality following radical cystectomy and extended pelvic lymphadenectomy from a single high volume center, Quek et al.[51] noted an overall mortality rate of 2%. Of note, this rate dropped to 1% in the last 10 years of this series. This is comparable with other large cystectomy series employing a standard lymph node dissection[52,53,54,55]. Obviously, certain factors may affect the ability to perform a pelvic lymph node dissection, such as prior pelvic radiotherapy, surgery, and individual anatomy. The urologic surgeon should consider such factors on an individual basis when determining the feasibility and extent of lymphadenectomy.

Brössner et al.[56] specifically evaluated this question in a contemporary retrospective review of 92 patients. They found no difference in short-term complications for patients undergoing an extended lymphadenectomy, although the operative time was on average 63 minutes longer. In a single center study, Poulsen et al.[42] did not experience any increase in morbidity when they changed to an extended template after their first 68 patients. The rate of lymphoceles and lymphedema was evaluated in a series of 447 patients. In patients with <16 lymph nodes removed there was a 2% incidence of lymphocele and lymphedema formation versus 1.1% in patients with ≥16 lymph nodes removed[35]. Despite the fact that no prospective trials exist comparing the outcomes of a standard versus an extended pelvic lymph node dissection with radical cystectomy, there does not appear to be any increased morbidity associated with an extended lymphadenectomy in appropriately selected patients.

## PROGNOSIS AND THERAPEUTIC BENEFIT IN BLADDER CANCER

The routine use of an extended pelvic lymph node dissection in bladder cancer is a debated topic despite reports to suggest improved clinical outcomes[57]. Poulsen reported a small 5-year recurrence-free survival benefit of 6% between patients who underwent an extended versus a standard lymph node dissection (25 vs. 14 lymph nodes removed). This difference did not reach statistical significance for the entire cohort, although for the subset of patients with ≤ T3a primary tumors the survival benefit did reach significance[42]. A large, single-center study from Germany involving 447 patients demonstrated a significant improvement in 5-year disease-free survival in patients who had greater then 15 lymph nodes evaluated (65% vs. 51%)[35]. Herr et al.[58] noted that evaluation of <10 versus ≥ 10 nodes resulted in decreased overall survival, 44% versus 61%. In the review of the SEER database, Konety et al.[32] demonstrated improved cancer-specific survival if patients had greater then 3 lymph nodes removed, but the greatest risk reduction was seen in patients with 10-14 lymph nodes removed. In 2002, Herr et al.[34] reported their single-center experience on the effect of the number of lymph nodes removed in both node negative and node positive patients. The study included 322 patients who had not received either neoadjuvant or adjuvant therapy and who had been followed for 10 years. Five-year overall survival was improved in node negative patients when ≥ 8 compared to <8 lymph nodes were removed, 82% versus 41%. A significant overall survival was seen in node positive patients as well. The cutoff for removed lymph nodes in this group was ≥ 11 or <11, with survival rates of 44% versus 20%, respectively.

Skinner[59] reported his experience with a series 153 patients, of which 36 were found to have lymph node metastases. He found that patients with 1-5 versus >5 positive lymph nodes experienced a 5-year overall survival of 46% and 12.5%, respectively. Stein et al.[60] recently updated this group's experience using a cut-off of ≤ 8 or >8 positive lymph nodes. The 10-year disease-specific survival was 40% and 10%, respectively. A study from Memorial Sloan Kettering of 686 patients demonstrated a 10-year disease-specific survival of 42% and 22% for patients who were pathologically N1 and N2, respectively[38]. In a large contemporary series from Egypt, Abdel-Latif et al.[37] reported 3-year overall survival at 59%, 32%, and 7% for patients with 1, 2-5, and >5 positive lymph nodes following radical cystectomy. Clearly, the surgeon cannot control the number of lymph nodes that are positive, but a diligent excision of all lymphatic tissue draining the bladder will increase the probability that metastatic disease will be identified and removed.

Several authors have evaluated the concept of lymph node density (number of positive nodes divided by the total number removed). Herr[61] evaluated a cohort of 162 lymph node positive patients who were followed for 7.5 years. There was a significant improvement in 5-year survival for patients with <20% versus >20% positive lymph nodes, 64% versus 8% respectively. This concept was independently reported by Stein et al.[60] in a series evaluating the outcomes of 244 patients with lymph node metastases. They determined that patients with <20% had a 10-year recurrence-free survival of 43% versus 17% for patients with >20%. Abdel-Latif[37] evaluated lymph node density using the following cutoffs; <10%, 10-20%, and >20%. The 3-year overall survival was 56%, 39%, and 16%, respectively.

## CONCLUSION

Currently, pelvic lymphadenectomy is the most sensitive and accurate modality available to detect occult nodal disease in both prostate and bladder cancer patients. Extended pelvic lymph node dissection will remove more lymph nodes than the "standard" lymphadenectomy. Whether or not the removal of more lymph nodes truly makes a difference in survival remains controversial.

The onus is on the urologic surgeon to provide each patient the best chance at cure from prostate and bladder cancer. Despite the availability of prognostic nomograms, it may be difficult to accurately select patients in whom a pelvic lymph node dissection can be safely omitted. With multiple factors contributing to node counts (many of which are beyond the control of the surgeon), one should focus on the dissection template to assure an adequate resection. As there appears to be little difference in morbidity compared to a more limited dissection, it is the authors' belief that a meticulous extended lymphadenectomy be considered in all patients undergoing radical prostatectomy and radical cystectomy.
